# Predictability of Persistent Frequent Attendance in Primary Care: A Temporal and Geographical Validation Study

**DOI:** 10.1371/journal.pone.0073125

**Published:** 2013-09-05

**Authors:** Frans T. Smits, Henk J. Brouwer, Aeilko H. Zwinderman, Marjan van den Akker, Ben van Steenkiste, Jacob Mohrs, Aart H. Schene, Henk C. van Weert, Gerben ter Riet

**Affiliations:** 1 Department of General Practice, Academic Medical Center, University of Amsterdam, Amsterdam, The Netherlands; 2 Department of Clinical Epidemiology, Biostatistics and Bioinformatics, Academic Medical Center, University of Amsterdam, Amsterdam, The Netherlands; 3 Department of General Practice, CAPHRI School for Public Health and Primary Care, Maastricht University, Maastricht, The Netherlands; 4 Department of General Practice, Katholieke Universiteit Leuven, Leuven, Belgium; 5 Department of Psychiatry, Academic Medical Center; University of Amsterdam, Amsterdam, The Netherlands; Federal University of Rio de Janeiro, Brazil

## Abstract

**Background:**

Frequent attenders are patients who visit their general practitioner exceptionally frequently. Frequent attendance is usually transitory, but some frequent attenders become persistent. Clinically, prediction of persistent frequent attendance is useful to target treatment at underlying diseases or problems. Scientifically it is useful for the selection of high-risk populations for trials. We previously developed a model to predict which frequent attenders become persistent.

**Aim:**

To validate an existing prediction model for persistent frequent attendance that uses information solely from General Practitioners’ electronic medical records.

**Methods:**

We applied the existing model (N = 3,045, 2003–2005) to a later time frame (2009–2011) in the original derivation network (N = 4,032, temporal validation) and to patients of another network (SMILE; 2007–2009, N = 5,462, temporal and geographical validation). Model improvement was studied by adding three new predictors (presence of medically unexplained problems, prescriptions of psychoactive drugs and antibiotics). Finally, we derived a model on the three data sets combined (N = 12,539). We expressed discrimination using histograms of the predicted values and the concordance-statistic (c-statistic) and calibration using the calibration slope (1 = ideal) and Hosmer-Lemeshow tests.

**Results:**

The existing model (c-statistic 0.67) discriminated moderately with predicted values between 7.5 and 50 percent and c-statistics of 0.62 and 0.63, for validation in the original network and SMILE network, respectively. Calibration (0.99 originally) was better in SMILE than in the original network (slopes 0.84 and 0.65, respectively). Adding information on the three new predictors did not importantly improve the model (c-statistics 0.64 and 0.63, respectively). Performance of the model based on the combined data was similar (c-statistic 0.65).

**Conclusion:**

This external validation study showed that persistent frequent attenders can be prospectively identified moderately well using data solely from patients’ electronic medical records.

## Introduction

Some patients visit their general practitioner (GP) relatively often. This frequent attendance is mostly defined as an age- and sex-adjusted attendance rate ranking in the top 10% within a time frame of 1 year [1], [2] Frequent attenders (FAs) are responsible for 39% of all face-to-face consultations of their GPs and persistent frequent attenders (those 1.6 percent who frequently attend during three consecutive years or more; pFA) are responsible for about 8% of face-to-face consultations. [3] Frequent attenders and, in particular, persistent frequent attenders have relatively many somatic, psychiatric and social problems [3].

Although longitudinal studies on frequent attenders are scarce, it is known that most frequent attenders frequently attend their GPs for a short period of time only [3]–[7]. It seems neither reasonable, nor efficient to target extensive diagnostic assessment, monitoring, and intervention at transient 1-year frequent attenders. However, patients who continue to attend frequently may require special attention, and potential effective interventions should probably be targeted at this group. Prediction of persistent frequent attendance may therefore be clinically useful if effective treatment of underlying medical problem and (thereby) prevention of this persistence is available. Scientifically a prediction model for pFAs may be useful to help select more homogeneous high-risk populations for future randomized trials or support efficient subgroup analysis [8], [9].

A review on the effects of (mainly psychiatric) interventions on morbidity and attendance rates has shown conflicting results. [10] One out of a total of 5 trials showed that a depression management program improved quality of life and the number of depression-free days of patients frequently attending the GP during two years. [11], [12] None of the included trials showed an effect on healthcare utilization of frequent attenders. All trials, except the one mentioned above, included patients that attended frequently during just one year. [12] Therefore, some negative findings may have been due to strong regression to the mean and spontaneous ‘recovery’, making it difficult to detect any effects. A more recent Spanish study in 1-year FAs showed that a 15 hours' training of GPs, which incorporated biopsychosocial, organizational, and relational approaches resulted in a reduction of attendance rates (mean number of annual contacts in the intervention group 13.1 against 19.4 in the usual care group) [13].

Using information that was readily available in GPs’ electronic medical records (EMR), we developed a prediction rule to help GPs identify, among 1-year frequent attenders, those at extremely low or high risk of becoming persistent frequent attenders (see table 1). [14] With the indicators available in the EMR presented in our previous study, our rule was modestly effective in selecting those at risk of becoming persistent frequent attenders (AUC 0.67; CI 0.64–0.69).

**Table 1:** Original prediction rule [14]: Associations between five predictors and persistent frequent attendance (pFA), the dependent variable.

Predictor	(adjusted) Odds ratio	95% confidence interval limits
Age¶	0.99	0.98–1.00
No. of active problems¶	1.13	1.05–1.22
Any chronic somatic illness	1.55	1.25–1.93
Any psychological problem	1.72	1.30–2.27
Average monthly No. analgesics prescriptions: none	1	Reference
1–4	1.77	1.41–2.23
>4	2.06	1.59–2.66

Based on 3045 observations; 470 pFAs (dependent variable = 1);

¶ modeled as a continuous variable; All other variables were modeled as dummies.

Since many diagnostic indices perform worse in a different population, (external) validation in a different primary care population is warranted before the use in clinical practice is advocated. [15] In this study we temporally (another time frame) and geographically (another area) validated our previously derived prediction model for pFA-ship using information solely from GPs’ EMR and looked for opportunities to improve it with extra patient information.

Because they are more prevalent in pFAs and theoretically likely to increase persistence of frequent attendance, we added 4 extra variables (sex, presence of medically unexplained symptoms, prescriptions of psychoactive drugs and antibiotics) to the original model and tested it for improvement on the 3 cohorts (including original cohort) [3], [16], [17].

We also explored building a more robust model based on the combined data of all three datasets.

## Methods

### Ethics Statement

The study was conducted according to the Dutch legislation on data protection (Ministry of Justice, the Netherlands). Ethics approval was provided through the Medical Ethics Review Committee of the Academic Medical Center of the University of Amsterdam (letter W 12_259#12.27.0295), stating that “the Medical Research involving human subjects Act (WMO) does not apply to this study and that an official approval of this study by our committee is not required”.

### Patient Population

To validate our prediction rule we used two primary care cohorts:

1. *Temporal validation;* Six primary healthcare centres in Amsterdam and Diemen provided data for the second cohort. These centers participate in the GP-based continuous morbidity registration network of the Department of General Practice, Academic Medical Center - University of Amsterdam. [14] This cohort was an enlarged version of our original cohort (4,032 vs. 3,045 adult patients) in a more recent time frame (2009–2011). See our original article for more details [14].

2. *Geographical and temporal validation;* All primary healthcare centres of the Eindhoven Corporation of Primary Health Care Centres in Eindhoven provided data for the first cohort. These centers participate in the GP-based Study on Medical Information and Lifestyles Eindhoven (SMILE) of the Department of General Practice of Maastricht University. [18] The patients studied were of average socioeconomic level, of more western descent, and slightly older than the general Dutch population. This cohort consisted of 5,462 patients who were FA in 2007 and we followed them to 2009.

In both networks, EMR data are extracted for research purposes. The participating GPs use a problem-oriented registration method. This study used the numbers of face-to-face consultations with the GPs, the lists of current medical problems as registered and coded by the GPs using the International Classification of Primary Care (ICPC), and a selection of prescriptions of all patients [19].

### Selection of One-year Frequent Attenders and Persistent Frequent Attenders

In all cohorts, frequent attenders were defined as those adult patients whose attendance rates ranked nearest to the top 10th centile of their age group (15–30, 31–45, 46–60, ≥61 years) separate for men and women. [1], [2] Persistent frequent attenders were defined as those patients who were both registered and frequently attending during 3 consecutive years. Only face-to-face consultations with GPs (consultations in the office and house calls) were included. Consultations with other practice staff were excluded because, in the practices involved, such consultations are mostly initiated and planned by GPs or their staff and cover mainly chronic disease programs.

### Definition and Extraction of Predictor Information

We considered potential predictors of persistent frequent attendance which were easily obtainable in all three cohorts (see table 2). In the problem-oriented approach to medical record keeping, a patient may have a list of current medical problems, also called *a problem list*. In the Netherlands, a current medical problem is defined as:

**Table 2:** Comparison of the three databases.

		A’dam I#	A’dam II	SMILE
Time period		2003–2005	2009–2011	2007–2009
Patients n^1^		28,680	40,320	54,620
Frequent attenders n		3,045	4,032	5,462
Persistent Frequent Attenders n (%)		470 (15%)	629 (16%)	1,107 (20%)
‘Lost to follow up’ n (%)		436 (14.3)	608 (15.1)	199 (3.6)
Mean age (SD^2^)		42.6 (18.2)	47.9 (18.5)	45.9 (18.8)
Females n (%)		1,566 (51)	2,179 (54)	2,640 (48)
Consultations of pFAs^3^ (mean n/year)^4^		10.2	11.8	7.7
Problems on the problem list				
Active problems (Frequent Attenders) n (SD)		2.03 (2.16)	2.68 (2.70)	1.70 (1.55)
Any chronic somatic illness n (%)		1,259(41)	1,906(47)	2,768 (50)
Any psychological or social problem n (%)		690 (23)	1,028 (26)	2,781 (51)
Any Medically Unexplained Symptoms n (%)		391 (13)	610 (15)	98 (2)
Prescriptions of analgesics mean n/month (%)	0	1,484(49)	1,889 (49)	2,759 (51)
	1–4	1,061 (35)	1,446 (36)	2,703(50)
	>4	500 (16)	597 (15)	
Any psychoactive medication n (%)		938 (31)	1,230 (31)	1,775 (33)
Prescriptions of antibiotics mean n/month (%)	0	1,976 (65)	2,374 (59)	3,120 (57)
	1–2	814 (27)	1,172 (29)	2,342 (43)
	>2	255 (8)	486 (12)	

1 n indicates number.

2 SD indicates standard deviation.

3 pFAs indicates persistent Frequent attenders, frequent attenders during 3 years.

4 Respectively in 2005 (A’dam I), 2011 (A’dam II) and 2009 (SMILE).

# A’dam indicates the Amsterdam I cohort.

Any disease or complaint which, according to the GP, needs continuing medical attention or monitoring and/or
Any disease or complaint present for more than 6 months and/or
Recurrent medical problems (more than 4 episodes per half year) [20].

Every problem on this list was coded by the GPs using the ICPC. [19] The prevalence of each medical problem was calculated at the end of the year. Medically Unexplained Symptoms (MUS) were defined according to Robbins *et al*. [21].

See the supporting file (table S1) for a list of the selected ICPC-codes.

### Statistical Analysis

#### Validation of model to predict persistent frequent attendance

The prediction model, to be validated in the present analysis, has been derived in our previous study using (bootstrapped) multivariable logistic regression analysis. This model included the variables: age (Odds ratio (OR) 0.99 per year), number of active problems (OR 1.13 per additional problem), presence of any chronic somatic problems (OR 1.55), any psychological problems (OR 1.72) and the monthly number of analgesic prescriptions (>4: OR 2.06). [14] See Table 1.

We first validated the original prediction model through the assessment of its discrimination (predictive values, c-statistic and corresponding 95% confidence intervals) and calibration (slope and corresponding 95% confidence intervals, and Hosmer Lemeshow plots and tests comparing predicted versus observed risk). A calibration slope of 1 indicates that predicted probabilities match observed risks perfectly (100%), a slope <1 indicates overprediction [22]–[24]. We repeated this analysis for an extended model with four extra variables.

#### Recalculation of the prediction model in the combined cohorts

Recalculation of the original model was performed using all pooled subjects from the three cohorts (update cohort, n = 12,539). Regression coefficients were obtained using logistic regression with persistent frequent attendance as the dependent variable and age, number of active problems, any chronic somatic problem, any psychological problem and the monthly number of analgesic prescriptions as predictors. Analyses were performed using SPSS for windows, version 20.

## Results

### Prediction of Persistent Frequent Attenders

**Figure 1:**
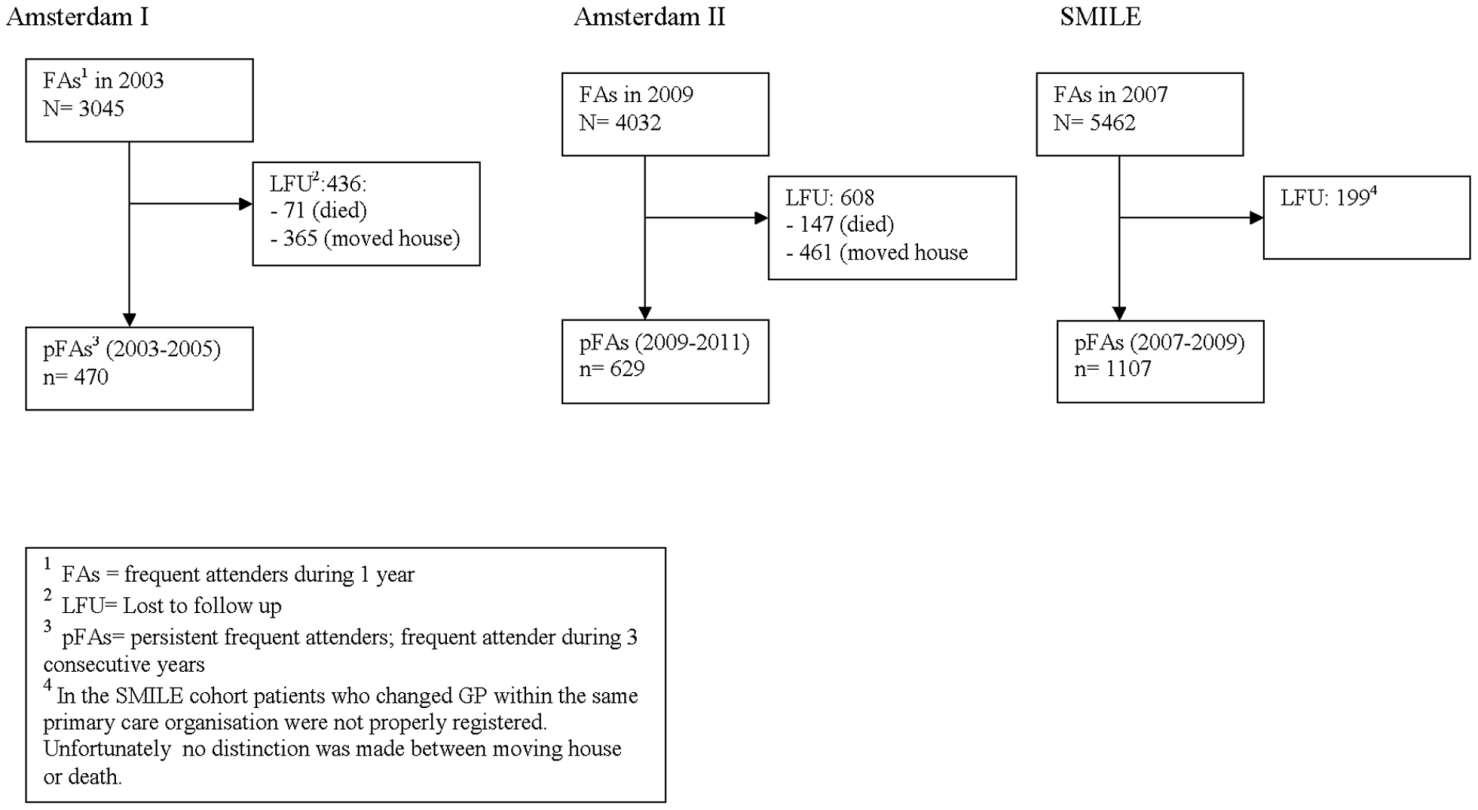
Flow chart of the 3 databases.

Table 2 and figure 1 show the general characteristics of the three cohorts. The persistent frequent attenders comprised 15–20% of all registered adult patients in all cohorts. Compared with both Amsterdam cohorts, patients in the SMILE cohort were relatively more often pFAs, had less loss to follow-up, fewer active problems, more psychological problems and fewer medically unexplained symptoms. In SMILE the mean number of prescriptions for analgesics and antibiotics was higher, but lower for psychoactive medication. In the SMILE cohort patients who changed GP within the same primary care organization were not registered as having moved house. Unfortunately, no distinction was made between moving house or death. We corrected for loss to follow (LFU) up by measuring the prognostic index with and without correction for LFU. The results did not materially change. See the supporting file (Table S2).

Table 3 shows the results of the original prediction rule in the Amsterdam II cohort and the SMILE cohort. Using the original regression weights with shrunken coefficients the c-statistics were 0.67 (95% confidence interval (CI) 0.64–0.69) in the original database, 0.62 (CI: 0.60–0.65) in the Amsterdam II cohort and 0.63 (CI: 0.61–0.65) in the SMILE cohort. Re-estimation of the regression weights did not change the results much (table 3). As expected in low-prevalence settings, negative predictive values were high, but all other indices were of moderate size.

**Table 3:** Discrimination and calibration on external validation of the original prognostic index to the Amsterdam II and SMILE cohorts.

	Amsterdam I		Amsterdam II		SMILE	
number ofFAs/pFAs^a^	3,045/470		4,032/629		5,462/1107	
*Using the original regression weights* #						
C-statistics(95% CI)^b^	0.67	0.64–0.69	0.62	0.60–0.65	0.63	0.61–0.65
Positive predictive value	0.27		0.22		0.27	
Negative predictivevalue	0.90		0.89		0.86	
*Re-estimation of the regression weights*						
C-statistics(95% CI)	0.67	0.64–0.69	0.64	0.61–0.66	0.63	0.62–0.65
Positive predictivevalue	0.27		0.22		0.27	
Negative predictivevalue	0.90		0.89		0.86	
*Adding 34 other predictor variables* c						
C-statistics(95% CI)	0.67	0.65–0.70	0.65	0.62–0.67	0.65	0.63–0.66
Positive predictivevalue	0.26		0.23		0.26	
Negative predictivevalue	0.90		0.89		0.88	
*Calibration of the original prognostic index* d						
Slope (SE)	0.99	0.08	0.656	0.07	0.83	0.06

a (p)FAs: (persistent) Frequent Attenders: frequently attending patients during 1 and 3 years, respectively.

b Concordance statistics (95% confidence interval).

c Female sex; any medically unexplained symptoms; any psychoactive medication; mean monthly number of prescriptions of antibiotics.

d Ideally, the slope should be 1, which indicates perfect calibration of predicted and observed risks. Values <1 indicate overoptimism (shrinkage), that is, high risks are overestimated, while low risks are underestimated.

# using a model with shrunken coefficients of the original model (shrinkage coefficient 0.993).

Sensitivity, specificity, likelihood ratios and positive and negative predictive values were calculated at the value where their sum was maximal (Q-point of the ROC curve).

**Figure 2:**
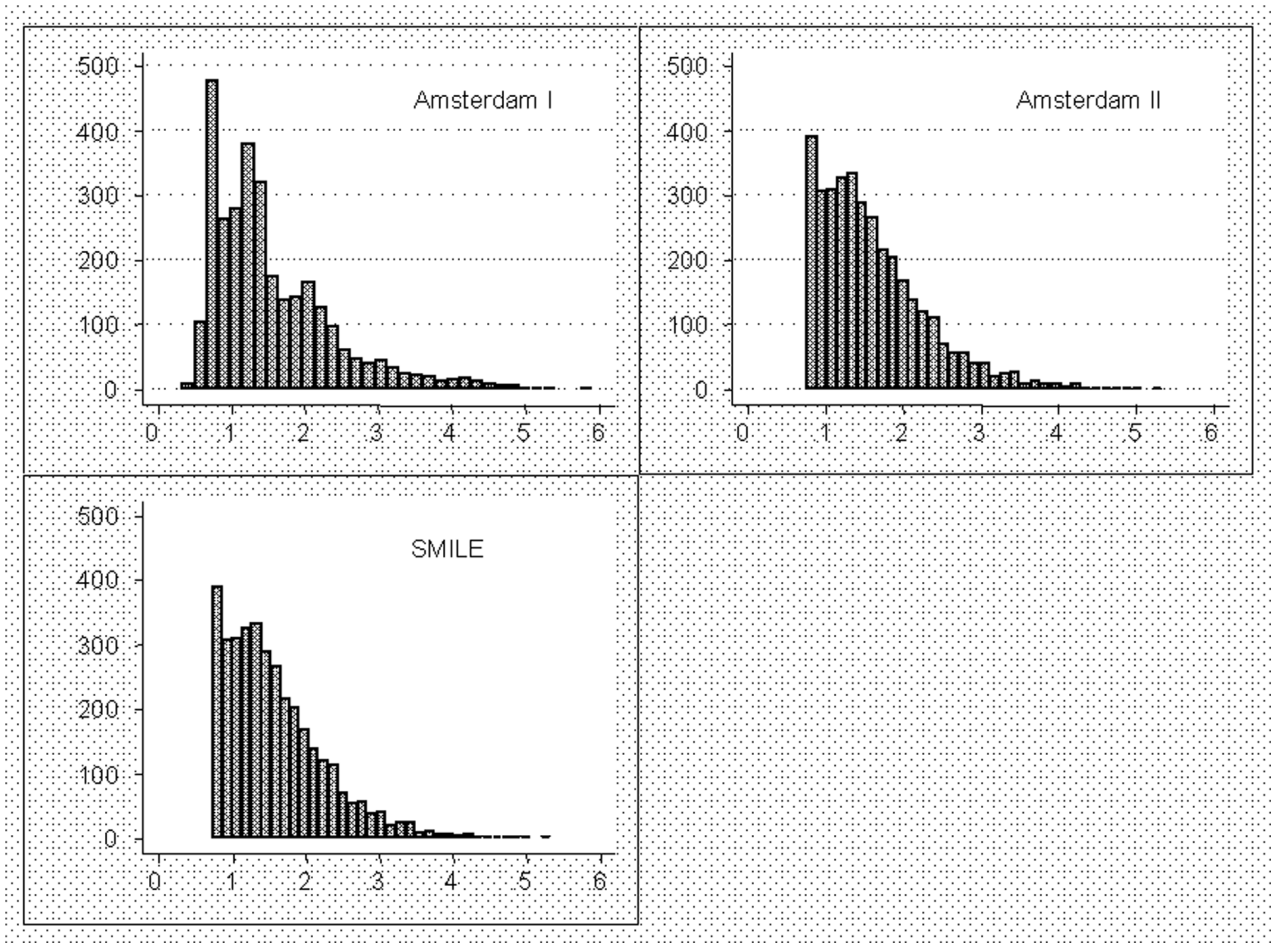
Histograms showing the predicted values based on the model predictions for the three cohorts, Amsterdam I (the original (derivation) cohort) and the two external validation cohorts. Histograms showing the predicted values based on the model predictions for the three cohorts, Amsterdam I (the original (derivation) cohort) and the two external validation cohorts, Amsterdam II and SMILE. The graphs illustrate the slight overoptimism of the original model and the shrinkage of the distributions, that is, the tails of the AMC II and SMILE cohort distributions are slightly closer to the center and predicted values smaller than 7 percent or greater than 54% no longer occur on external validation. Y-axes are frequencies.

Figure 2 shows the predictive values (predicted probabilities of becoming pFA) based on the prediction model in the three cohorts. Predictive values on external validation (AMC II and SMILE) lay between 7.5 percent and 54 percent, slightly more conservative than those in the original cohort (3.3 and 59%).

The effect of clustering on the health center level was negligible with intraclass correlations of 0.02–0.06 and almost no change of the regression weights. See the supporting file (Table S2).

Adding three predictors (any MUS, any psychoactive medication and the mean monthly number of antibiotic prescription) hardly changed the performance (c-statistics Amsterdam II 0.65; CI 0.62–0.67; SMILE 0.65; CI 0.63–0.66) (see table 3).

**Figure 3:**
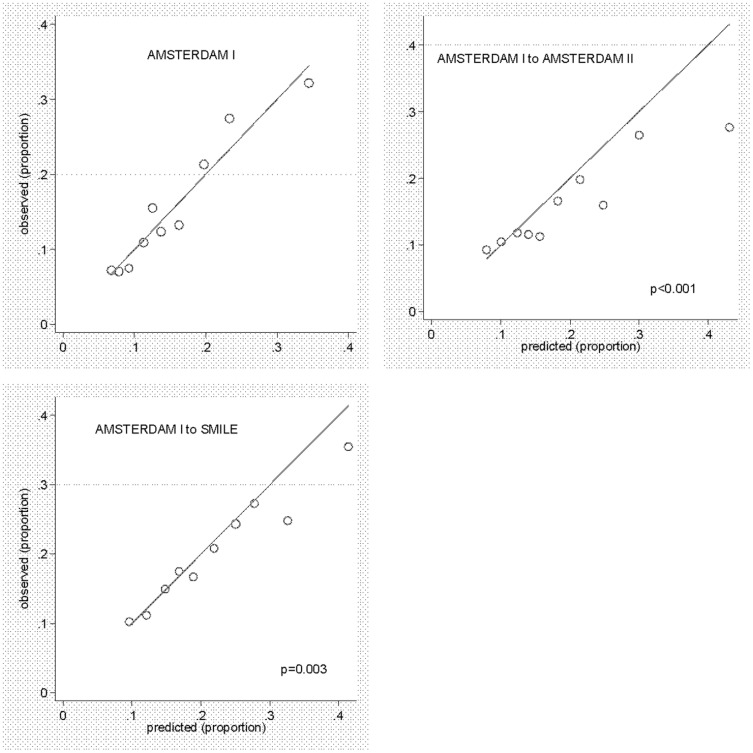
Hosmer Lemeshow plots: Observed versus predicted risk for persistent frequent attendance. In these Hosmer-Lemeshow calibration plots, each circle represents the observed mean probability of becoming a persistent frequent attender (pFA) within a decile of patients after all patients were ordered from lowest to highest predicted probability. As usual, the Hosmer Lemeshow calibration plot in the top left hand corner shows a good match between predicted and observed risks in the derivation cohort (Amsterdam I) as all circles are close to the diagonal of perfect calibration. On external validation in the Amsterdam II cohort (top right hand graph), eight out of ten predicted values were higher than those observed and those in deciles 5, 8 and 10 (extreme right hand circle) in particular. On external validation in the SMILE cohort, predicted probabilities matched the observed ones well, except for the two highest deciles, 9 and 10. The small p-values are also partly caused by the large sample size so that small mismatches become statistically significant. Note that the vertical distance to the diagonal represents the mismatch between predicted and observed pFA probabilities.

The Hosmer Lemeshow plots showed modest calibration with underestimation of the probability to become pFA (see Figure 1, upper right circles which tend to fall below the line y = x). This was confirmed by the small p-values indicating large discrepancies between predicted and observed risks. (See fig. 3).

### Updated Prediction Model

We updated the prediction model using all pooled patients of the three cohorts (12,539 patients). Pooling the three data sets and fitting a new model did not materially lead to important improvements (c-statistic 0.65; CI: 0.63–0.66).

## Discussion

### Summary of Main Findings

External validation in time and place of an existing prediction model for persistent frequent attendance in primary care showed that its discrimination remained stable while calibration was reduced for the higher predictions in particular. Model extension with three plausible predictors not previously included hardly improved model performance.

### Strength and Limitations of the Study

Important strengths of this study are the size and the longitudinal character of the datasets and the experience of the participating GPs in recording and coding the problem lists. [20] The SMILE cohort had relatively little loss to follow-up (mostly because fewer patients ‘moved house’ because of registration limitations) and more pFAs. Knowing the way GPs and practice staff cooperate in the Netherlands, replacement of GP consultations by practice staff consultation (off-utilization bias) will be very limited. [25] Prescriptions are extracted from the electronic medical record and reflect the number of actual prescriptions. Prescription data in general practice may generally be considered to be of higher quality than diagnosis-oriented data. [26] The higher prescription rates of analgesics and antibiotics and the lower rates for psychoactive medication are therefore difficult to understand and may reflect the different patient populations in both cities and/or different prescription habits of the local GPs. Wennberg showed that everyday clinical practice is characterized by wide geographical variations that cannot be explained by illness severity or patient preference. [27], [28] The present retrospective study was based on routinely collected data and therefore reflects everyday general practice in the Netherlands. Because we wanted to predict patient behavior, we only used GP-patient consultations and not planned monitoring consultations for chronic diseases with other primary care staff.

However, there are also some limitations. First, there are differences between the Amsterdam and SMILE cohort which may have influenced the predictive performance of our rule. In general, within a General Practice Research Network, one distinguishes four categories of factors to explain morbidity and prescription differences: “healthcare system”, “methodological characteristics of the network”, “general practitioner”, and the “patient”. These factors and sub-factors are often interrelated. [29] The differences between the cohorts in Amsterdam and Eindhoven may partly be explained by methodological differences (the shorter existence of SMILE (fewer active problems) and coding agreements (fewer MUS, but more psychological codes in SMILE)), different populations of general practitioners (shorter experience in coding problems in SMILE) and patient factors (more stable population; more females in SMILE). However, socio-demographic characteristics of populations cannot explain the differences in morbidity estimations among these cohorts [30].

The problem lists may suffer from overreporting (by not removing resolved problems, for instance, depression) and underreporting (for instance personality disorder). This may have diminished the predictive power of “any psychological problem”. Moving out of practice was a reason for exclusion, as follow-up of these patients was not possible. Compared with SMILE the loss to follow-up rate was higher in Amsterdam, but in the original study this did not result in selection bias. [14] Finally, the presumed higher registration quality in these academic networks may diminish the generalizibility of a prediction rule derived in these networks to other practices.

Formal external validation of prediction models while important is still scarce. If the original prediction model had had excellent performance, one may expect worse performance on external validation. External validation usually reveals the so-called over-optimism of the original model. [31] Our original model performed moderately well and its discrimination performance remained largely intact, although the predicted risks did not match the observed risks very well for the more extreme risk predictions. Using shrunken coefficients of the original model had a very limited effect and there was no sign of selective loss to follow-up. Finally, accounting for clustering within health care center had little impact.

### Comparison with Existing Literature

The few longitudinal studies about frequent attendance showed that attendance rates tend to regress to the mean over time, with only 20–30% of frequent attenders continuing to attend frequently in the following year [4]–[6], [32] However, these studies of persistent frequent attendance used different definitions of frequent attenders and lacked the power to detect factors associated with transient frequent attendance becoming persistent. Vedsted found that psychological distress, as measured with two psychometric scales, increased the risk of future daytime frequent attendance of adult patients in family practice. [16] Another, small, prospective study (85 primary healthcare patients of working-age) detected as risk factors for persistent frequent attendance female sex, body mass index above 30, former frequent attendance, fear of death, alcohol abstinence, low patient satisfaction, and irritable bowel syndrome. [33] Smits et al showed that with the indicators currently present in Dutch electronic medical records, a rule performed modestly in selecting those more likely to become persistent frequent attenders [14].

### Implications for Clinical Practice

After validation and updating the existing rule only predicts persistence of frequent attendance moderately. For clinical use this rule has some significance to predict which 1yFAs have more risk to become a persistent FA. We are currently prospectively following a cohort of 623 frequent attenders for three years and hope to be able to improve predictions by incorporating better and more patient-based information, such as socioeconomic status, body mass index, health anxiety/illness behavior, depressive complaints and anxiety. This has to be weighed against increased time and costs to collect such information.

### Implications for Future Research

The model may be useful to select populations for RCTs with a higher likelihood of becoming pFA. In addition, prediction models can be used in RCTs to characterize the trial arms at baseline in a multivariable way, thus enhancing the assessment of baseline comparability. [9], [34] Finally, subgroup analyses using the scores from prediction models may serve to move beyond multiple univariable subgroup analyses [9], [35].

### Conclusion

Prediction of persistent frequent attendance using data solely from EMRs currently available in the Netherlands may be moderately helpful in identifying those patients at high (or low) risk of becoming persistent frequent attenders. Better predictors are needed to improve prediction.

## Supporting Information

Table S1 **Selected problems and diseases with ICPC-code^1^.**
(DOCX)

Table S2 **Effect of loss to follow-up and clustering on the health centre level on the prognostic index.**
(DOC)
