# Supplementary material for: Predictability of Persistent Frequent Attendance in Primary Care: A Temporal and Geographical Validation Study
Source: PLoS One. 2013 Sep 5;8(9):e73125. doi: 10.1371/journal.pone.0073125 (PMC3764153; doi:10.1371/journal.pone.0073125)
Supplement: Table S1 — Selected problems and diseases with ICPC-code1. (DOCX) [file pone.0073125.s001.docx]

**Table S1. Selected problems and diseases with ICPC-code^1^**

| **Disease group** | **ICPC- code** | **Disease** |
| --- | --- | --- |
| Diabetes | T90 | Diabetes mellitus type 1 and 2 |
| Chronic cardiovascular disease | K74  K75  K76  K77  K78  K82  K83  K86  K87  K89  K90  K91  K92 | Angina pectoris  Acute myocardial infarction  Other and chronic ischemic heart disease  Heart failure  Atrial fibrillation/flutter  Pulmonary heart disease  Heart valve disease, non-rheumatic**^2^**  Hypertension, uncomplicated  Hypertension with involvement of target organs  Transient cerebral ischemia  Cerebrovascular accident; stroke  Atherosclerosis excluding heart and brain  Other arterial obstructive/ peripheral vascular disease |
| Chronic respiratory problems | R70  R91  R95  R96  R97 | Tuberculosis of respiratory organs  Chronic bronchitis/ bronchiectasis  Emphysema/Chronic Obstructive Pulmonary Disease  Asthma  Allergic rhinitis; hay fever |
| Psychological/Psychiatric problems   1. Anxious feeling 2. depressed feeling   3) addictive behaviour | All P  P01  P74  P09  A-Y27  A26-Y26  A25  B25  K24  K25  X23/Y25  X24/Y24  X25  P03  P76  P15  P16  P17  P18  P19 | All Psychological-codes  feeling anxious/nervous/tensed  Anxiety disorder/anxiety state  Concern sexual preference  Fear of other disease of various tracts  Fear of cancer of various tracts  Fear of death  Fear of AIDS  Fear of heart attack  Fear of hypertension  Fear of venereal disease  Fear of sexual dysfunction  Fear of genital cancer  Feeling depressed  Depressive disorder  Chronic alcohol abuse  Acute alcohol abuse  Tobacco abuse  Abuse of medicines  Drug abuse |
| Medically unexplained symptoms (MUS) | L01  L02  L03  L04  L18  N01  N02  A04  P06  P20  T03  T07  T08  P04  R02  K04  N17  R21  N06  D09  D11  D08  D12  D01  D93 | Neck symptoms /complaints (excluding headache)  Back symptoms/complaints  Low back complaints without radiation  Chest symptoms/complaints  Muscle pain/ fibromyalgia  Headache  Tension headache  General weakness/tiredness/chronic fatigue  Disturbance of sleep/insomnia  Disturbance of memory, concentration  Loss of appetite  Weight gain  Weight loss  Feeling/behaving irritably  Shortness of breath, dyspnea  Palpitations/ aware of heartbeat  Vertigo/Dizziness  Symptoms / complaints of the throat  Other sensitive disturbance /abnormal involuntary movements  Nausea  Diarrhea / Loose bowels  Flatulence/gas pain/belching  Constipation  Generalized abdominal pain/cramps  Irritable bowel syndrome |

**^1^** International classification of primary care

**^2^** Not otherwise specified
