# Supplementary material for: Predictability of Persistent Frequent Attendance in Primary Care: A Temporal and Geographical Validation Study
Source: PLoS One. 2013 Sep 5;8(9):e73125. doi: 10.1371/journal.pone.0073125 (PMC3764153; doi:10.1371/journal.pone.0073125)
Supplement: Table S2 — Effect of loss to follow-up and clustering on the health centre level on the prognostic index. (DOC) [file pone.0073125.s002.doc]

**Table S2.** Effect of loss to follow-up and clustering on the health centre level on the prognostic index.

|  | Amsterdam I | Amsterdam II | SMILE |
| --- | --- | --- | --- |
| Deviance of the normal Logistic Regression model | 2476 | 3046 | 5320 |
| Ln(OR)^1^ of the prognostic index (SE) ^2^ | 0.993 (0.084) | 0.663 (0.071) | 0.835 (0.062) |
| Ln(OR) of the prognostic index corrected for Loss to follow-up (SE) | 1.028 (0.080) | 0.694 (0.067) | 0.848 (0.062) |
|  |  |  |  |
| deviance of the multilevel model | 2467 | 3029 | 5198 |
| Ln(OR) of the prognostic index multilevel model (SE) | 0.994 (0.085) | 0.707 (0.073) | 0.909(0.064) |
| variance practice effect | 0.072 | 0.102 | 0.222 |
| Intraclass correlation coëfficiënt | 0.0214 | 0.0301 | 0.0632 |

^1^ indicates natural logarithm of the odds ratio

^2^ SE indicates Standard Error
